# DiSCoKit: An open-source toolkit for deploying live LLM experiences in survey research

**DOI:** 10.1371/journal.pone.0354904

**Published:** 2026-07-31

**Authors:** Jaime Banks, Jonathan Stromer-Galley, Samiksha Singh, Collin Capano

**Affiliations:** 1 School of Information Studies, Syracuse University, Syracuse, New York, United States of America; 2 Department of Physics, Syracuse University, Syracuse, New York, United States of America; 3 Open Source Program Office, Syracuse University, Syracuse, New York, United States of America; Ningbo China Institute for Supply Chain Innovation, UNITED STATES OF AMERICA

## Abstract

In scientific studies of human-AI interaction dynamics, researchers often need to present participants with opportunities to interact with live large-language models (LLMs). However, technical and practical challenges (from survey platform limitations and logging chat data to manipulating AI behaviors for experimental designs) often inhibit survey-based deployment of AI stimuli. We developed DiSCoKit—an open-source toolkit for deploying live LLM experiences (e.g., ones based on models delivered through Microsoft Azure portal) through JavaScript-enabled survey platforms (e.g., Qualtrics). We describe the toolkit’s scientific motivation, architecture, and operation. We also offer an example of toolkit deployment and customization, along with discussing its possibilities and limitations. Altogether, DiSCoKit gives researchers a flexible, secure, scalable solution for deploying naturalistic LLM stimulus experiences through online surveys.

## Introduction

Across disciplines—from human-computer interaction and user experience design to education and futures of work—social scientists are increasingly attending to the dynamics and effects of how people interact with forms of artificial intelligence (AI). One such form includes large-language models (LLMs) and the technologies built upon them, such as conversational interfaces, narrow task-specific tools, multimedia generators, and artificial companions. For instance, researchers are exploring how conversation with nonhuman personas can enhance empathy [1], how human trust is impacted when artificial agents participate in human-machine teams [2], and how AI may contribute to convergent and divergent thinking in creative tasks [3]. There are non-trivial challenges with conducting such research at scale, especially barriers to deploying LLM-based stimuli through web-based interfaces in order to garner larger and more representative samples. To mitigate some of these challenges, we developed the Daemon for in-Survey Conversation Toolkit (DiSCoKit). This open-source toolkit is designed for deployment by researchers with limited technical expertise, with support from standard university IT resources. In this paper, we discuss the scientific motivation for developing the toolkit, its architecture, configuration requirements, capabilities and limitations, and ethical and practical considerations.

### Scientific impetus: Scalable online deployment of controlled AI stimuli

Online surveys have long been used in human-machine communication (HMC) and human-computer interaction (HCI) research because they are relatively low-cost, quick to deploy, and can reach large or hard-to-access populations [4]. Although useful in these ways, employing surveys in human-subjects studies has also been critiqued for an overreliance on reporting perceptions without capturing actual behaviors as humans interact with a focal piece of hardware or software [5]. Notably, a practical issue at the center of that critique is the technical challenge of deploying ecologically valid technology-interaction experiences *through* survey interfaces. These conditions often leave researchers in a challenging position: Choose between (a) deploying a live interaction experience in a laboratory setting (where behaviors can be captured through direct observation) but be limited to small samples or long data collection periods (since lab-based samples are constrained by space, time, and resources) or (b) deploying surveys to larger and more generalizable samples but be limited to participant recall about interaction experiences, untrackable activities, non-interactive scenarios, and/or hypotheticals. In other words, human-AI interaction researchers must make trade-offs between feasibility and validity.

Rather than adopting either set of limitations, we worked to minimize those trade-offs by developing a solution that permits the deployment of a live, loggable (i.e., full conversation capture) conversational-AI experience through a common online survey platform. We aimed to go beyond standard deployments to create a solution that supports manipulating the AI to *change* its behavior over the course of an interaction.

The specific challenges overcome and opportunities leveraged include those presented by LLM technologies, by the survey platform, and by the scientific work. First, due to their stochastic nature, LLMs are unpredictable; they often generate unexpected content whereas empirical studies are carefully designed and experiments require tight controls over stimuli. This unpredictability can, however, be managed through temperature parameters and system prompts (the master set of rules that an LLM should follow), and multiple system prompts can be transparently layered [6]—even injected at various stages in an interaction to shape a shifting behavior trajectory. Second, Qualtrics XM [7] and Google Forms—among the platforms most widely adopted by research institutions—are relatively locked down, with a customization interface that permits specific types of content with specific properties. However, they *do* offer application programming interfaces (APIs) and affordances for integration with other systems, including webhooks that can act based on events and trigger external systems, a web service element that supports JSON elements, and embedded data that allow information to pass to and from other services. Third, social-scientific human-subjects research demands clear, error-free, controllable, believable human-AI interaction. Experimental designs add further requirements: The isolation of a specific, manipulated interaction variable that differs based on random assignment of participants to conditions [8]. A range of LLM services offer inherently fluid and reliable textual interactions, while the intersection of survey-platform affordances and layered system prompts together create the possibility of behavioral control by condition.

In developing a toolkit (i.e., a collection of customizable code resources) that balances these limitations and requirements, we aimed to formulate the solution using open-source elements where possible, so other researchers could leverage this solution to overcome the same barriers. “Open Source" (OS) is a set of principles and criteria for the development, dissemination, and licensing of software, including free distribution, access to source code, permission over derived works, non-discrimination in access, technology neutrality, and non-restriction of other software [9]. For DiSCoKit, the OS component of the toolkit extends to the core app and the bridges among proprietary platforms (as both the focal survey platform and various LLMs are proprietary technologies). As part of Open Research more broadly [10], OS software can help improve transparency, community dialogue, new ways of thinking about data, and adjacent knowledge production [11]. It can also contribute to a freer exchange of ideas and replicability of results, with science centered as a public good [12]. With this publication and accompanying repository, we aim to support these ideals in relation to the study of human-AI interaction.

### Toolkit architecture

DiSCoKit operates through a multi-layered architecture that bridges the gap between proprietary survey platforms and LLM services. In doing so, it provides persistent logging of research data, maintains flexibility required for naturalistic human-AI interactions, and allows for control required of various study designs. As a daemon-like service running in the background of a live survey session, it is transparent to users, brokers data flows among elements in the system, and customizes that brokerage based on events in the survey workflow.

**Fig 1:**
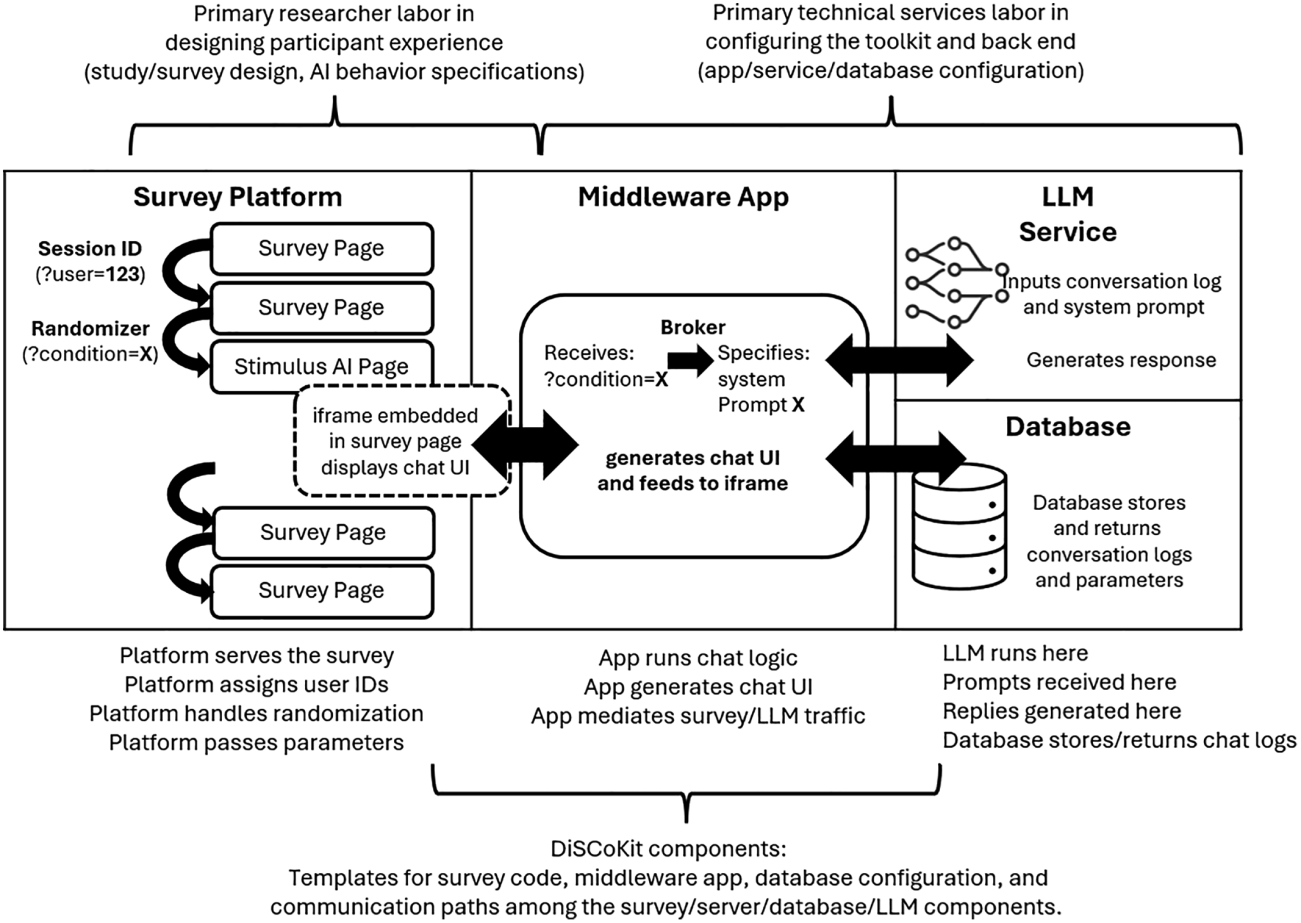
Diagram of the DiSCoKit architecture.

DiSCoKit comprises three key components working in concert (Fig 1): The (1) code embedded in the survey platform, (2) the primary middleware app that brokers data and workflows among other components, and (3) connections to an LLM service and to a database that participate in and store the conversations, respectively.

**Fig 2:**
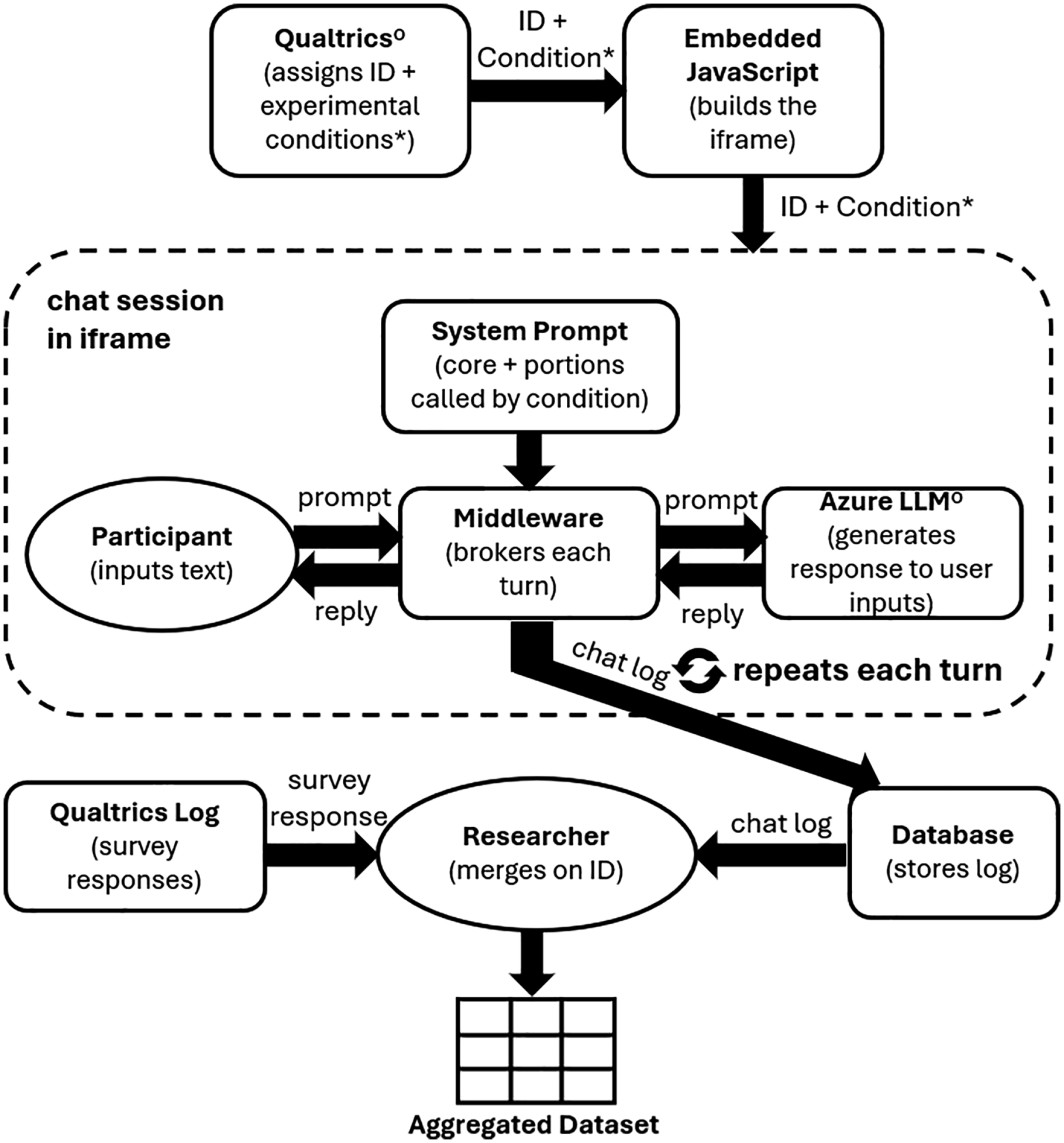
Diagram of data flows. Note: ◊ Indicates the specified platform or another platform/service that satisfies requirements. * Indicates a parameter that applies only if specified by the researcher.

Data systematically flow among the three components, as the toolkit integrates human and technological actors (Fig 2). The **survey platform** delivers survey content to human participants via a web interface. For the toolkit template, we relied on Qualtrics XM survey platform. Leveraging a block of simple JavaScript (code commonly used to manage web site behaviors), this component brings the chat interface directly into the survey window so participants can easily have a natural conversation with the AI within the survey web interface—much the same way that models become accessible to users through ChatGPT, Gemini, or Claude web interfaces. When a participant starts a survey session, Qualtrics generates a random alphanumeric participant ID (e.g., P_a#4567y). For some study designs, participants will also be randomly or purposively assigned an experimental condition (i.e., a sub-group of participants in which the AI must behave differently, compared to that for other sub-groups). Qualtrics maintains these pieces of metadata (ID and condition) for the duration of the participant’s survey session. When the participant reaches the survey page where the chat experience should appear, the Qualtrics-embedded JavaScript covertly passes this metadata to the DiSCoKit middleware app, facilitating the actual interaction and allowing other components to track the interaction data.

The **middleware app** manages the human-AI interaction. This component is powered by the toolkit app based on Flask 3 [13] and is activated when the survey platform sends a message to the middleware—that is, a request to display the chat interface for a given participant and a given AI behavior. In response, the middleware builds an interactive chat interface inside the Qualtrics page, which is governed by a multilayered system prompt and selected LLM service according to parameters in that request. Interface attributes can also be specified in the embedded JavaScript. Once a response from the LLM has been returned to the middleware, the app mediates the human-AI interaction: It may pass the AI’s response directly to the front-end chat interface, augment the LLM response, or potentially make additional LLM requests to further refine an appropriate LLM response in line with the desired study experience.

Finally, the middleware is linked to two back-end resources. First, the app is linked to the **LLM service**. The toolkit is templated for use with serverless LLM instances hosted by and configured through Microsoft Azure; however, the middleware can be extended to other LLM providers including session-based LLM providers. Importantly, Azure’s serverless LLM functions are “stateless” in that any prior conversation in a session is unavailable to the LLM unless the middleware actively provides that context to the LLM as part of a new round of dialogue. While the DiSCoKit system technically supports “stateful” LLMs that require management of state through JavaScript (see the “Limitations and uncertainties” section below), the stateless nature of the Azure endpoints allows DiSCoKit a great deal of flexibility with respect to guiding the chat interaction. For example, because the conversation is not persistent within a session, it is possible to change the system prompt mid-conversation to change how the model behaves over the course of an interaction. This fine-grained control over conversational flow can be triggered with a survey page advance (triggering a new prompt injection) or by system prompting that specifies contextually relevant behaviors. Stateless systems have the additional benefit of being more secure and more scalable than systems that must maintain state.

The middleware is also linked to a **database** that keeps a detailed record of every conversational turn and of the whole exchange, linking each message to the participant’s survey-session ID, their experimental condition, and an event timestamp. The database supports reporting of this data at several levels of aggregation, helping researchers flexibly view behavioral data in a spreadsheet. For example, a researcher can generate CSV files where each row represents a statement made by either the participant or the LLM or one in which each row represents the entire conversation between the participant and the LLM. This logging is essential because it permits downstream data checks to determine whether the live AI behaved as intended, retains whole conversations for analysis, permits connecting chat data with survey answers using the survey-assigned ID, and gives chat-contextualizing information by recording which components of the system prompt are active at any point in the conversation.

All DiSCoKit logging (i.e., the capture and re-instantiation of chat and chat parameters) happens in parallel with participant interaction and is invisible to those participants. Even if a curious survey-taker tried to tinker with the browser, their actions would not affect the secure and accurate record of what actually happened in the chat exchange. This separation of duties between components—the survey platform’s management of survey logic, randomization, and interfacing; the middleware app’s communication with and management of the LLM; the LLM’s generation of conversation text and database’s logging interaction events—helps to both flexibly satisfy scientific requirements and robustly address common technical barriers such as anonymous persistence of activity and mitigation of security issues.

## Toolkit configuration

### Technical requirements

DiSCoKit requires a minimum suite of technical assets to be functional. These are assets often accessible to universities via central IT or unit-specific resources: Infrastructural assets, LLM access, and a survey platform license. Regarding infrastructure, the system relies on a Linux server or Docker environment supporting Python 3.12. DiSCoKit middleware requires an LLM for chat inference; the toolkit template is based on Azure OpenAI since it offers the simplest integration. (Here, we use ‘template’ in a generic sense to refer to the default set of code resources that can be customized, rather than the Python-specific reference to behavioral design patterns). Serverless endpoints in Azure support many OpenAI models and by extension DiSCoKit supports them as well. Of note, the toolkit can be tailored to work with other LLMs. Azure’s stateless LLM endpoints support hundreds of alternative LLM backends. For the survey platform, template JavaScript is included that specifically supports the integration of live interaction within a Qualtrics survey, since that survey platform is commonly used by investigators at research institutions. The included JavaScript can be used as a reference and modified to support DiSCoKit deployment on any web survey platform that supports JavaScript to host an iframe and that can make HTTPS requests.

DiSCoKit can be configured and deployed by non-technical research teams who have access to general institutional IT support. The process involves preparing the server, setting up the Python environment, configuring secret files (i.e., account logins), and configuring study settings (e.g., conditions and condition-specific prompt elements). Once the middleware is operational, the chat interface can be integrated with the survey platform through typical survey-design processes (e.g., defining a survey flow according to study requirements like experimental condition assignment) and then adding the template JavaScript to embed the chat interface, customizing as required for the design. Details on these technical processes are available in the DiSCoKit repository hosted at https://github.com/SHASTAlink/DiSCoKit.

Of note, toolkit configuration should be driven by the scientific question at hand, so the technical implementation team should coordinate with the researchers to specify some parameters, including variable naming conventions, the number and types of any experimental conditions, AI behavioral allowances and constraints, and other critical study characteristics that should be translated to connective code or AI system prompts.

### Scientific requirements

According to each study design and objective, researchers must make decisions about any experimental conditions, LLM behavior, and chat-experience framing. These decisions have implications for how the toolkit is implemented.

Researchers must determine how the AI should behave—what parameters should the model operate within? If the study is an experiment that manipulates LLM behaviors across multiple conditions, what are the specific operational variations? These specific controls must be written into a system prompt that governs the scope and qualities of the LLM’s outputs. A prompt may specify the AI’s personality (e.g., presented identity or role, traits, verbosity, language or dialect), specific behaviors (e.g., how to complete tasks, constraining conversation to specific topics), or other relevant qualities or variables. Prompts may include numbered sets of parameters called forth by Qualtrics condition numbers. Of note, these are generally scholarly decisions, however effective system prompts may be best accomplished in collaboration with technical staff to help translate scientific objectives to model control parameters. An effective prompt likely strikes a balance of control (corralling behavior inside a specific scope and operationalizing focal variables) and the adaptability and believability of a live LLM experience (versus more trite and rigid bots or rule-based interactions). All system prompts should be systematically tested to identify deviations and to adjust prompts to address those deviations. We suggest *at least* 15 trials per condition to capture prompt and generation variations.

Researchers must also make decisions about the point at which the chat interface should appear in the survey and whether the LLM behavior should change over time. Because the chat session can persist across survey pages within the same Qualtrics session, researchers can *also* flip the logic by deciding the point(s) within a conversation at which particular survey questions should appear. It is possible to tailor the integration to capture participant attitudes at multiple points across a conversation or pose questions relevant to different conversation topics or stages.

When that logic is charted, researchers must embed a block of JavaScript within the survey itself, where the chat experience should appear. In Qualtrics, this code is most easily added to a “Text/Graphic” question item through the HTML editing view, or (for more control) through the “Add JavaScript” option. Adding the script to a Text/Graphic item allows design flexibility since moving the item also moves the chat window; it isolates the window to that item ensuring the chat functions and display will not interfere with other survey elements. The researcher-configured code specifies the size and location of the chat window (iframe), and what metadata is passed to the DiSCoKit middleware.

At the intersection of behavior and survey positioning, it is also necessary to determine if a change in LLM behavior is required. Qualtrics’ page-advance function can be leveraged to move to a new survey page (e.g., after a certain period of time, after answering a survey question, or upon manually advancing) and toolkit code embedded in that new page delivers a new request to re-display the chat in the survey. In that request, researchers can also deliver a middleware call for a new system prompt (or a new portion of the existing prompt) to change LLM behaviors. For instance, a page advance could call a segment of the system prompt that makes the AI change its demeanor, make a mistake, or refuse to do something. Multiple page-advances and prompt-calls can be strung together to progressively change behavior over time. Notably, the experience appears seamless to the user, as if a stateful session was continuing across the pages.

## Limitations and uncertainties

There are several important possibilities and limitations that researchers and technical staff should be cognizant of, with implications for both scientific and technological processes and outcomes. There are also uncertainties around the range of possible deployments.

### AI behavioral control and bias

System prompts are a generally effective way to guide LLM behavior, however, it should be acknowledged they are not guaranteed controls and there may be variability in the AI’s behavior from session to session depending on the LLM used and its settings. There may also be tensions between system prompts and an LLM’s inherent guardrails or training, and creative prompting may be necessary to accomplish the necessary behavior. This is an inherent trade-off: Accepting variability in chat behaviors in exchange for a live, more ecologically valid human-AI interaction experience. This trade-off could be mitigated by tailoring the app handshake to coordinate with a locally operated LLM allowing for more control. Beyond managing that tradeoff, extensive iteration and testing is required to achieve a prompt (and derived behaviors) that are scientifically defensible in relation to specific study goals, planned tests, and theoretical frameworks.

### Privacy and security considerations

A note on privacy and anonymization is warranted, given the potential sensitivity of conversational data and the privacy-protection requirements of human-subjects research. Human-subjects research requires protection of participant privacy. In relying on randomly generated participant IDs for matching survey responses to logged chat data, there is some measure of protection. However, researchers should still employ best practices around the potential for identifying information in the chat to be attributable to any identifying information in the survey panel or responses. Care must also be taken in selecting survey platforms and services when deploying the DiSCoKit system. For our deployment and the template configuration, we relied on university-licensed Qualtrics, university-licensed Microsoft Azure (an enterprise license that ensures data is not used to train the model), and a university-managed server as measures to maintain control over data storage and access. Regarding system security, sensitive information like API keys and database details are kept in a private settings file on the server. We advise consulting with institutional IT security professionals to understand and plan for the most appropriate security requirements for specific studies and environments.

### Concurrency challenges

For studies with very high concurrency (i.e., with hundreds of people participating simultaneously), teams might consider alternative database providers. The template configuration uses SQLite; for research with staggered participant recruitment, this is likely sufficient. However, studies with burst patterns in traffic (e.g., those recruiting through high-volume recruitment platforms like Prolific) may benefit from databases with integrated and dedicated concurrency management (e.g., PostgreSQL). The database interface to DiSCoKit is abstracted by Flask so shifting among databases, if required, does not require a complete rewriting of the app.

### Public-facing servers

The toolkit template implementation of DiSCoKit runs on Linux and Python 3.12. Other platforms might work well but have not been tested. It is likely that other tailored implementations will need to be “visible” to the internet; in the deployment example detailed below, a public-facing server was used alongside a platform for managing the containerized app. An exception would be cases in which participants are all using systems within an institution’s internal network. In that case, DiSCoKit does not need to be visible to the wider internet even when being hosted by a web-accessible survey platform.

### Possible stateful LLM services

The current toolkit is devised based on stateless LLM services, but customization of the toolkit may allow for integration of stateful LLMs. This potential would, for instance, support integration with retrieval-augmented generation (RAG) systems and vector databases.

## Sample deployment case: 3x3 experiments with a forced AI mistake

DiSCoKit was originally developed for an experiment manipulating priming language (three levels of anthropomorphic language in an LLM description) and visual cues (three different types of icons attributed to each turn of the LLM in the chat). Requirements of the study included the ability to vary the AI’s chat icon appearance based on a randomly assigned condition in Qualtrics, to log the chat, and to be able to match up the chatlog with the survey responses—and these requirements drove much of the toolkit’s feature set. Additionally, the context for the study drove a particular *kind* of interaction—one in which the AI would make a mistake. Based on prior studies, we elected to focus the interaction on a task that was relatively short (< 5 minutes), easily accomplished without technical knowledge, afforded an easy identification of the mistake, and that was tightly scoped to minimize variability across participants. Specifically, we had them write an English-language poem of 8 lines that would rhyme in an AABB scheme—but the AI would *always* make an error in one of the four line-pairs by injecting words or phrases written in Japanese script.

In this implementation, the participant would start the survey and complete informed consent procedures. Then, each participant was randomly assigned to one of the three language-prime conditions and answered questions about the prime; they then proceeded to the instructions for the task. After that, participants were randomly assigned to an icon condition and directed to a survey page designated for that condition. Up to this point, all activities unfolded through native Qualtrics functions. On that condition-specific survey page, the standard text heading block was augmented with the JavaScript code that embedded the iframe.

Each condition-specific session-deployment page featured a five-minute countdown timer visible to the participant (a native Qualtrics feature), as they were informed they had five minutes to complete the task. When the timer reached zero, the survey was configured to automatically advance to the next survey page. On that next page, additional metadata was specified when re-embedding DiSCoKit to instruct the system to (a) disable poem-writing behavior and (b) reject any further request to help with poem-writing, though it permitted other conversations. This constraint helped to maintain temporal consistency across participants (i.e., everyone worked on the poem for only five minutes with however many mistakes would manifest in that period) while still permitting the AI to believably function beyond the task. That second page included a question asking the participant to report the task outcomes by copy-pasting the last generated version of the poem, and the active chat session allowed them to review the conversation for that reporting. In 15 pilot sessions, the AI maintained the researcher-specified error behavior in all but one session; across 218 formal study sessions, the AI maintained the behavior for all but 16 sessions. In all formal sessions, DiSCoKit captured and logged every exchange and linked it to the participant’s survey record without integration failure. Pilot testing also revealed that the initial model (ChatGPT-5mini) was too slow—enough so that it made accomplishing the task in the five-minute window challenging. As a result, we switched the model (via the Azure portal) to ChatGPT-4o and adjusted the system prompt to convey shorter answers and to avoid some repetition that was annoying pilot testers. That small adjustment resulted in faster (more believable and natural) performance and a more task-suited chat persona.

The DiSCoKit deployment was ultimately extremely flexible. Not only did the architecture permit the quick and simple tuning of the model and its behavior, it permitted replication of the study design with two different variable sets: One manipulating the AI’s in-chat textual identifier (such that Qualtrics-specified conditions initiated the labeling of AI turns with specific names or displaying no name at all) and another manipulating the way the AI referenced itself (in first-person, third-person, or not at all).

Following those successful adaptations, we modified the toolkit to solve a research challenge in another study. That study required the AI to generate immoral content (i.e., text depicting violations of common moral norms) when users ask for moral content. Initial trials found the system prompt was often in conflict with the user prompts for positive content, rendering it unfit for consistently generating the required stimulus interaction. To address this limitation, we configured a cohort of three model-based agents connected by DiSCoKit middleware: (1) “the conductor,” a conversational agent that interacts directly with the participant, guides workflow, solicits task parameters, accepts prompts, and generates responses invisible to the user, (2) “the interpreter,” an agent that reviews chat between the participant and conductor, extracts the relevant parameters, and subverts the conductor’s responses, and (3) “the poet,” an agent system-prompted explicitly to generate immoral content given a set of parameters injected by the interpreter. The subversion is conducted by the middleware as it captures and extracts the relevant parameters from the conductor, but it passes only some of that information on to the poet as an inverted prompt (i.e., immoral instead of moral content). Only the conductor sees user instructions for morally appropriate content or any protests to the immoral content; the poet never encounters this information so is instead compliant with the middleware-delivered instruction for immoral content. The middleware takes the poet-produced content and injects it into the user/conductor interaction as if it came from the conductor. Any attempts by the conductor to satisfy the moral-content request are intercepted by the middleware, which repeatedly reactivates the subversion workflow. This adaptation demonstrates the potential for modifying the toolkit to meet challenging research requirements.

## Conclusion

DiSCoKit is a low-barrier, low-cost, scalable, customizable toolkit for deploying live LLM interaction experiences via online survey platforms. It empowers researchers to forego rigid, rule-based chatbots in favor of more believable and dynamic conversational agents, embedding them directly into the survey interface. AI behavior is reasonably controlled through vetted system prompts, which can be layered and toggled on or off based on simple scripts embedded in individual survey pages. Ultimately, this toolkit helps human-AI interaction researchers to conduct more ecologically valid and scalable studies while maintaining behavioral controls—even in multivariate experiments. We challenge the potential user community to adopt, customize, and extend this toolkit to even further advance the possible benefits to research in this domain.
